# Species distribution model transferability and model grain size – finer may not always be better

**DOI:** 10.1038/s41598-018-25437-1

**Published:** 2018-05-08

**Authors:** Syed Amir Manzoor, Geoffrey Griffiths, Martin Lukac

**Affiliations:** 1 School of Agriculture, Policy and Development, University of Reading, Reading, UK; 2 Department of Geography and Environmental Science, University of Reading, Reading, UK; 3 Faculty of Forestry and Wood Sciences, Czech University of Life Sciences Prague, Prague, Czech Republic

## Abstract

Species distribution models have been used to predict the distribution of invasive species for conservation planning. Understanding spatial transferability of niche predictions is critical to promote species-habitat conservation and forecasting areas vulnerable to invasion. Grain size of predictor variables is an important factor affecting the accuracy and transferability of species distribution models. Choice of grain size is often dependent on the type of predictor variables used and the selection of predictors sometimes rely on data availability. This study employed the MAXENT species distribution model to investigate the effect of the grain size on model transferability for an invasive plant species. We modelled the distribution of *Rhododendron ponticum* in Wales, U.K. and tested model performance and transferability by varying grain size (50 m, 300 m, and 1 km). MAXENT-based models are sensitive to grain size and selection of variables. We found that over-reliance on the commonly used bioclimatic variables may lead to less accurate models as it often compromises the finer grain size of biophysical variables which may be more important determinants of species distribution at small spatial scales. Model accuracy is likely to increase with decreasing grain size. However, successful model transferability may require optimization of model grain size.

## Introduction

Species distribution models (SDMs) are becoming increasingly important in predicting spatial patterns of biological invasions, identification of hotspots for early detection and informing management of invasive species^1^. SDMs relate the presence/absence records of species to relevant environmental variables and subsequently project modelled relationships across geographical space using gridded layers of environmental data, producing a map indicating areas of potential species distribution^2^. One of the key features of gridded data is the ‘grain size’ – a term describing the geographical representation (spatial resolution) of the map layers. Grain size of predictor variables strongly affects the interpretation of biogeographic characteristics of modelled species^3^. Use of smaller or finer grain size allows for a more accurate representation of the effect of local environmental conditions and biotic interactions in model prediction^4^.

The challenge in using smaller grain size in SDMs is finding the optimum balance between data quality, data availability, and model performance^5^. Grain size represents the geographical space unit which contains all the information on characteristic attributes of the study area^6^. A decrease in grain size enhances the details of the landscape by sharpening the features it contains and by making the rare land use types in the landscape more prominent and distinguishable^7^. Conversely, coarse grain size of predictor variables in SDMs negatively affects the delineation of habitat features in a landscape, a feature of critical importance to modelling species presence. Selection of grain size and its relationship with habitat features is a crucial factor in SDM based studies^3,7–9^. Most literature to date reports on species distribution models built at a grain size of 1 km, a fact recently subjected to some scrutiny and critique^7,10^. Earlier observations indicate that the use of 1 km grain size may be too coarse to generate reliable SDM outputs^7^, especially for studies at small spatial scales. The challenge therefore, is to establish the threshold grain size at which predictor variables correctly describe local conditions and biotic interactions which play an important role in defining species’ range^11^.

The choice of grain size in SDM studies is sometimes based on data availability^12^ rather than relevant factors like species’ ecology and spatial scale of study. A review of more than 200 SDM-based research papers concluded that the choice of variables is ‘frequently opportunistic’ and that the majority of the studies, instead of making a tailored choice of variables, rely on a standard set of 19 bioclimatic variables^13^ which are available at a minimum of 1 km grain size. In a complementary analysis designed to provide an overview of current practice, we reviewed 59 recent SDM based studies published in peer-reviewed journals in 2016–2017 (Supplementary data S1). We confirmed that the most frequently used variables in MAXENT based ecological modelling studies are indeed, the 19 bioclimatic variables available from the ‘Global Climate Data’ (www.worldclim.org). We found that 55 out of the 59 studies selected the above-mentioned bioclimatic variables as input. Of these 55 studies 34 had used additional biophysical variables such as topography and land cover. These biophysical variables are available at a grain size as 100 meters or less. Since the grain size of all input variables in SDMs need to be harmonized, these biophysical variable are resampled to 1 km in when used in combination with the bioclimatic variables. Intriguingly, the results of 22 out of these 34 studies (which had both bioclimatic and biophysical variables) suggest that the variables critical to accurate species distribution prediction were the biophysical variables. Given the earlier argument that a finer grain size is more likely to improve model accuracy, the following speculation can be made: had these 22 studies not coarsened the biophysical variables – by avoiding the ‘customary’ choice of bioclimatic variables - this would have resulted in a more accurate prediction of species distribution. This speculation might appear to question the significance of bioclimatic variables in ecological models. It is a fact that bioclimatic variables are among the most frequently used variables in SDM based studies and rightly so as climate is a strong determinant of species’ distribution. However, an injudicious use of these variables without considering factors like species’ ecology, scale of study and optimal grain size is questionable^13,14^. Thus, we speculate that in many SDM based studies – especially at small spatial scale of study area - biophysical variables may be the more important ones and inclusion of bioclimatic variables in such cases may reduce the model accuracy.

One of the motivations for creating SDMs is to use them to predict the behaviour of a species colonizing new territory. Successful transferability of SDMs across space or time is extremely valuable in context of conservation planning. A basic assumption underlying SDMs is that the model is spatially and temporally transferable, i.e. the niche attributes are conserved across space and time^2^. Although the effect of grain size in SDMs is well documented^15–17^, its role in model transferability has not been put to sufficient scrutiny. There is evidence that although SDMs can accurately predict species distribution in the training area, their transferability to new areas is challenging due to numerous complex phenomena^18,19^. Among many factors, grain size has been reported as critical to satisfactory model performance and transferability^20,21^.

In this study we aim to test the role of grain size in SDMs both in the training and the transfer areas. Based on our review of literature, we speculate that over-reliance on easily available bioclimatic variables may lead to an unnecessary compromise on the grain size of critical variables, with potentially negative impact on the accuracy of model predictions and transferability. Specifically, we use a MAXENT modelling environment^22^ to model the distribution of *Rhododendron ponticum* (L.) in the Snowdonia National Park, Wales and then transfer the model to the Brecon Beacons National Park, Wales. The objectives of this study were to assess whether the decreasing the grain size improves model performance both in the training and the transfer area.

## Methods

### Species description

*Rhododendron ponticum* (L.) is an invasive plant species in the United Kingdom, having been introduced in the 18^th^ century as an ornamental plant. The main ancestor is reported to be the population of *R*. *ponticum* resident in the southern tip of Spain^23^. It is a perennial, evergreen shrub that generally invades woodlands^24^, although it has been shown to colonize other types of habitat too. The UK invasion by this shrub has been more intense in Western and North-Western areas of Britain, which are comparatively cooler and wetter. We chose Wales as the study region because it is one of the most affected regions of the UK to be impacted by invasions of *R*. *ponticum*. In this study, we trained the model on the dataset for the Snowdonia National Park in Wales^25^ and then transferred the model to the Brecon Beacons National Park. Given the scale of the invasion, it is clear that the current environmental, topographic and land cover conditions both in Snowdonia and the Brecon Beacons represent a range of conditions very suitable for *R*. *ponticum*.

### Species distribution modelling algorithm

We used MAXENT, a maximum-entropy based machine learning (presence/pseudo-absence) algorithm to model the distribution *R*. *ponticum* (L.) in Snowdonia National Park (the training area) and projected the model to the Brecon Beacons National Park (the transfer area). MAXENT predicts the probability distribution of a species on the basis of a given set of predictor variables and presence-only species occurrence data^22^. We selected MAXENT because, a) it does not require absence data^26^, b) it efficiently handles complex interactions between predictor and response variables^27^, c) being a generative model, it performs better than discriminative models when it comes to modelling with presence-only records, d) it can be run with both categorical and continuous data variables^28^ and, e) it efficiently transfers the model projections to another geographical area^2^. We used a reasonably large sample size^29^ and applied the recommended screening and verification of occurrence records.

### Presence records for model training and validation

For the training area (Snowdonia National Park), presence-only occurrence records of *R*. *ponticum* (L.) were obtained from COFNOD (Local Environmental Records Centre in Wales, UK). A dataset of 152 occurrence records was created by a continuous field observation campaign between 1981 and 2000. COFNOD has confirmed that the entire area of Snowdonia National Park was thoroughly surveyed by ground surveys and remote sensing tools, thus minimizing the possibility of sampling bias in the dataset. Consequently, we targeted the entire area of the National Park, generating 10,000 random background points to be selected during each replicate run of the model. We used independent occurrence records of *R*. *ponticum* (L.) in the Brecon Beacons National Park downloaded from the National Biodiversity Network (NBN) online database (www.nbnatlas.org), yielding 100 observations. Spatial uncertainty of all occurrence records was addressed by removing all duplicate or non-geo-referenced occurrence points. Occurrence data were spatially rarefied using SDM toolbox 2.0^30^ in ArcGIS 10.5 by eliminating all but one point present within a single grid cell of the predictor variable layers to avoid double counting of presence points.

### Selection of predictor variables

Predictor variables were selected in the following three steps. In the first step, two categories of variables were compiled. The first category of variables comprised the most frequently used variables in SDM studies: ‘Bioclimatic Variables’ (BCV). The second category of variables was based on our expert knowledge and a review of literature on the ecology of *R*. *ponticum* (L.): ‘Biophysical Variables’ (BPV). A set of 19 bioclimatic variables from ‘Global Climate Data’ (www.worldclim.org, version 2, 1970–2000), identified as the most commonly used suite of variables in SDM research^13^, formed the BCV category. An extensive review of literature and background knowledge of the *R*. *ponticum* ecology yielded the most important biophysical variables, namely; topography (altitude, aspect and slope), land cover and ‘distance from water channels’ which formed the BPV category^31–34^. Although Rhododendron is sensitive to many other ecological factors, we kept the BPV category to the above mentioned variables as these variables were the most pertinent ones at the current spatial scale of study.

In the second step of variable selection, a sub-set of variables from the BCV and BPV categories was created on the basis of grain size. The first variable set (VS-1) included both BCV and BPV categories, with the latter resampled to a 1 km grain size which is the smallest cell size of BCV. The second variable set (VS-2) comprised the BPV at 300 m grain size. The third variable set (VS-3) consisted of the same BPV but at 50 m grain size (Tables 1 and 2). The VS-1 represents the commonly reported approach used in SDM studies and thus can be considered the ‘control’ scenario. The VS-2 & VS-3 represent scenarios where bioclimatic variables are excluded to conserve the finer grain size of BPV. All input data layers were re-sampled using nearest neighbour (for discrete variables) and bilinear interpolation (for continuous variables) resampling techniques^35–37^. Collinearity among predictor variables negatively impacts the model due to the substantial amount of information shared between collinear variables. Therefore, collinearity in variables makes it difficult to correctly interpret the relative contribution or importance of variables in the model predictions^38^. A Pearson correlation coefficient cut-off of r ≤ 0.70 was applied to select the variablesfor use in the final model runs^38^ for all three sets of variables (VS-1, VS-2 and VS-3). The aim of this step was to reduce the negative impact of multicollinearity and to conform to statistical assumptions^39^.

**Table 1:** Predictor variables used in the study.

VS-1		VS-2		VS-3	
Grain Size 1 km		Grain Size 300 m		Grain Size 50 m	
Predictor Variable	Unit	Predictor Variable	Unit	Predictor Variable	Unit
Altitude	m	Altitude	m	Altitude	m
Aspect	°	Aspect	°	Aspect	°
Slope	°	Slope	°	Slope	°
Land Cover		Land Cover		Land Cover	
Distance from water channels	m	Distance from water channels	m	Distance from water channels	m
Mean Diurnal Range (monthly (max temp - min temp))	°C				
Isothermality (BIO2/BIO7)* 100					
Mean Temperature of Driest Quarter	°C				
Precipitation Seasonality (Coefficient of Variation)	C of V				

Acronyms VS-1, VS-2 & VS-3 refer to variable set 1, variable set 2 & variable set 3 respectively.

**Table 2:** Allocation of predictor variables to ‘variable categories’ and ‘variable sets’.

Predictor variable/s	Grain Size	Source	Variables Category	Variable Set
19 bioclimatic variables	1 km	WorldClim - Global Climate Data	BCV	VS-1
Distance from water channels	1 km	Edina Digimap Ordnance Survey	BCV	VS-1
Land Cover	300 m	Edina Digimap Ordnance Survey	BPV	VS-2
Topography (Altitude, Aspect, Slope)	300 m	Shuttle Radar Topography Mission USGS	BPV	VS-2
Distance from water channels	300 m	Edina Digimap Ordnance Survey	BPV	VS-2
Land Cover	50 m	Edina Digimap Ordnance Survey	BPV	VS-3
Topography (Altitude, Aspect, Slope)	50 m	Edina Digimap Ordnance Survey	BPV	VS-3
Distance from water channels	50 m	Edina Digimap Ordnance Survey	BPV	VS-3

Acronyms BCV, BPV, VS-1, VS-2 & VS-3 refer to Bioclimatic Variables, Biophysical Variables, Variable Set 1, Variable Set 2 & Variable Set 3 respectively.

### Model calibration

All three modelling scenarios were run in MAXENT (version 3.3.3a) with a default convergence threshold of 10^−6^ and with 5000 iterations to allow the model scope for convergence while reducing the risk of over- or under-predicting modelled relationships. We processed 25 model replications with a bootstrap resampling method randomly allocating 75% of the occurrence records in the training area for calibration and 25% for validation. To avoid dubious projections by the model, we used the ‘fade-by-clamping’ feature which removes heavily clamped (clustered) pixels from the final predictions^26^. Rest of the MAXENT calibration was set to default settings.

### Model Evaluation

#### Training area

Area Under the ROC (Receiver Operating Characteristic) Curve (AUC) was used to test the performance of the model against actual observations in the training area^27^. An AUC value of 0.5 shows that the model does not predict any better than random chance, whereas a value closer to 1 indicates a better performance of the model^40^. Permutation importance contribution was used to assess the relative significance of predictor variables. Fitted response curves were used to visually investigate the relationship between individual variables and predicted index of environmental suitability of *R*. *ponticum*. In addition to AUC, we used Continuous Boyce Index (CBI) as an additional assessment tool. The Boyce index requires presence data only and measures by how much model predictions differ from random distribution of observed presence across the prediction gradient. The continuous habitat suitability map is reclassified into *i* number of classes/bins. For each bin, Predicted and Expected frequencies are calculated. The Predicted Frequency is calculated by dividing the number of species’ occurrence points in the bin *i*, as forecasted by the model, by the total number of species’ occurrence points. The Expected Frequency is calculated by dividing the number of grid cells in bin *i* by the total number of grid cells. A P/E ratio is then calculated for each bin and a Spearman rank correlation coefficient rho (1-tailed test) evaluates if the ratio significantly increases as suitability increases (p < 0.05). The continuous values of the Boyce index vary between −1 and +1. Positive values indicate a model where predictions are consistent with the distribution of actual presence data, values close to zero mean that the model is no different from a random model and negative values indicate counter predictions (e.g. predicting no occurrence in areas where actual presence is recorded)^41,42^.

### Transfer area (Model transferability)

MAXENT produces continuous probability maps of habitat suitability in the selected geographical area. We used *R*. *ponticum* (L.) presence records in the Brecon Beacons National Park to evaluate model projection in the transfer area. Continuous Boyce Index (CBI) was used to assess how well MAXENT has transferred the model to a different geographical area^41,42^. CBI is considered one of the most appropriate metrics for assessing model predictions applied to presence-only datasets. There is some indication that CBI is a more reliable metric than AUC when it comes to validating model transferability to a different geographical area^43^.

### Data availability

Presence records of *Rhododendron ponticum* in Snowdonia National Park and Brecon Beacons National Park can be acquired from COFNOD (www.cofnod.org.uk) and NBN Atlas (www.nbnatlas.org) respectively.

## Results

**Figure 1:**
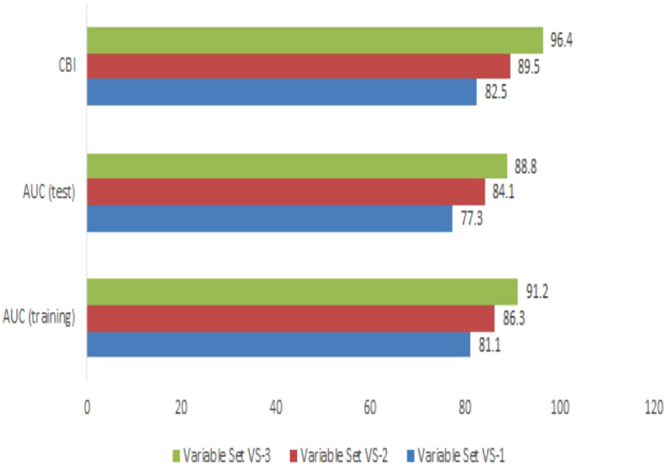
Area Under Curve (AUC) and Continuous Boyce Index (CBI) comparing prediction accuracy of Maxent-based models in Snowdonia National Park using three predictor variable sets at 1 km (VS-1), 300 m (VS-2) and 50 m (VS-3) resolution.

The AUC & CBI based evaluation of the three models in the training area, where each model used a different subset of predictor variables at different grain size, indicated variation in the degree of prediction accuracy. As shown in Fig. 1. AUC_train_, AUC_test_ and CBI values of VS-1, the variable set with the coarsest grain size are the lowest, indicating the least accurate predictions in the training area (Snowdonia). Variable sets VS-2 and VS-3, comprised of the same set of biophysical variables but at different grain size, indicate that the finer grain size is likely to yield better model predictions.

We used Continuous Boyce Index (CBI) to assess the transferability of the MAXENT models to an area not covered by the training dataset, in our case the Brecon Beacons National Park. The model comprising the VS-1 variables showed the poorest model transferability with a CBI value of 0.65. In comparison, the model based on the VS-2 dataset showed a high CBI of 0.90, while the third model based on VS-3 achieved a moderate CBI of 0.77. Analysis of the predictor variable contribution to model prediction (supplementary data S2) suggests that land cover and altitude were major contributors in all three models. Our results also suggest that the use of finer grain size improved model transferability (CBI value of Models VS-2 & VS-3 > VS-1). However, model transferability decreased at the finest grain size (50 m) of the predictor variables. Response curves for individual variables for all three modelling scenarios are provided in Supplementary data S3.

## Discussion

A number of studies have highlighted the fact that coarse grain size of predictor variables in SDMs may obscure effects of biotic interactions, small-scale heterogeneity of abiotic factors and micro habitat of species^44,45^. A review of 149-peer reviewed publications concluded that the choice of grain size is a highly neglected aspect in species distribution modelling and is a factor that significantly impacts modelling outcomes^12^.

### Model performance in the training area

The results from this study show that MAXENT model predictions in the training area are likely to improve with smaller grain size of predictor variables (AUC in the order of 50 m > 300 m > 1000 m grain size). The Snowdonia National Park is characterized by diverse topography, with altitude ranging from sea-level to above 1000 m over a relatively short distance. Altitude is one of the key factors affecting the invasive potential of alien species and the effect of altitude was shown to be most pronounced at fine grain size^46^. It has been claimed that too coarse a grain size in SDMs leads to spatial smoothing and thus obscures the connection between, for example, land cover types and species occurrence^47^. This occurs by homogenizing the dominant land types within a grid cell resulting in the loss of useful information for accurate modelling^48^. In accordance with this assertion, the accuracy of model predictions in our study improved with decreasing grain size of the predictor variables, possibly as the result of capturing small-scale ecological interactions critical for species distribution being maximized at a finer grain size^11,45,49^. In our case, the rugged topography of the area also affects factors such as soil physical and chemical properties, atmospheric humidity and wind speed/exposure over very short distances. With decreasing grain size, representation of these factors was more pronounced and improved model predictions. As grain size becomes finer, the number of mixed pixels decreases, leading to an increase in ‘distinct’ pixels which clearly separate different land cover, topographical or environmental units (or classes) and thus enables the algorithm to build more accurate species-habitat relationships^7^. This improvement becomes more relevant when the species being modelled is a habitat specialist. Since *R*. *ponticum* is considered one such species – in Wales it has a high preference for woodlands – better performance of models using small grain size data can be explained by improving representation of this community type.

As a habitat specialist, *R*. *ponticum* has repeatedly been shown to be strongly correlated with land cover type (Yang *et al*., 2013a). In Britain woodland is the most important land cover type in the context of *R*. *ponticum* invasion^23^, largely because of the availability of suitable micro-environments for seed germination^33^. For example, dead plant material and moss cover is critical to *R*. *ponticum* establishment^50^. Response curves in our study show that Forests are the most important land cover classes for *R*. *ponticum* distribution. Furthermores, *R*. *ponticum* is sensitive to topographic controls^51–53^. Response curves show that *R*. *ponticum* favors a northerly aspect for its establishment and growth as north-facing slopes at this latitude (Wales) are generally cooler, offering higher soil moisture and lower direct insulation intensity. Moreover, response curves suggest that *R*. *ponticum* distribution in Snowdonia is negatively correlated with slope. Shallow-slope areas are typically those with high soil moisture and nutrient availability, thus offering more favorable microenvironment for invasive species^54^. Distance from water channel was an important variable determining the habitat suitability of *R*. *ponticum*. This finding is compliments earlier studies suggesting that *R*. *ponticum* favors areas near water bodies^55^ primarily because soil in vicinity of water body is moist and often has dense vegetation. Many other invasive species have been reported to be negatively correlated with distance from water sources^56^.

### Model performance in the transfer area

After assessing model performance in the training area, the second goal of the study was to test the effects of grain size on the spatial transferability of the model. The results suggest that a coarse grain size (1000 m) produced the poorest model transferability while a medium grain size (300 m) resulted in the most accurate transfer of the model. The poor model transferability at 1 km grain size (CBI = 0.65) may be explained by the fact that key environmental factors, which in our case were land cover and topography, are ‘averaged out’ at coarser grain size both in the training and the transfer areas^44^. We expected the best model transferability when using data with the finest grain size. This was not the case; our transferred model had the best predictive power at medium grain size. A possible explanation is that Snowdonia National Park (training area) and Brecon Beacons National Park (transfer area) differ in the range and the character of topographical features. Since topography and land cover are best represented at small grain size, a discrepancy in the typography of landscape features between the two areas will negatively affect model transferability. Similarly, it has been shown that species occurrence data needs to be highly accurate when modelled at very fine grain size as any location^10,57^ errors in the survey data may impact model performance.

In this study the CBI value of the SDM transferred at 300 m grain size was 0.90, a reasonably accurate prediction but which leaves room for improvement. We tested SDM transferability under the assumption that abiotic factors are the principal controls on species distribution. However, the distribution of any species is also likely to be constrained by biotic interactions^58^. These biotic interactions vary between geographical regions, just as topography, land cover and climatic factors differ. Even though the training and transfer areas used in the study are similar, any difference in the nature of the biotic interactions limiting *R*. *ponticum* may have constrained the degree of model transferability^11^. In this context, this invasive species may have occupied only a subset of its potential niche in the invaded area so far, known as the realized niche. A species may fail to occupy the entire potential niche due to factors such as intra-species competition, dispersal limitation, scarcity of resources and other spatial limitations^59^. The distribution of species is linked to a framework known as ‘Biotic Abiotic Mobility’ (BAM)^60^ which describes the potential niche yet to be inhabited by a species in the ‘unfilled niche’^61^. Thus, correct identification of this unfilled niche may help to identify areas vulnerable for future invasion and may prove helpful in understanding the invasive behavior of species under study^62^. Our results suggest therefore, that for habitat specialists, model transferability across geographical space becomes highly sensitive to the grain size when the model training and transfer areas differ in environmental and ecological features.

**Figure 2:**
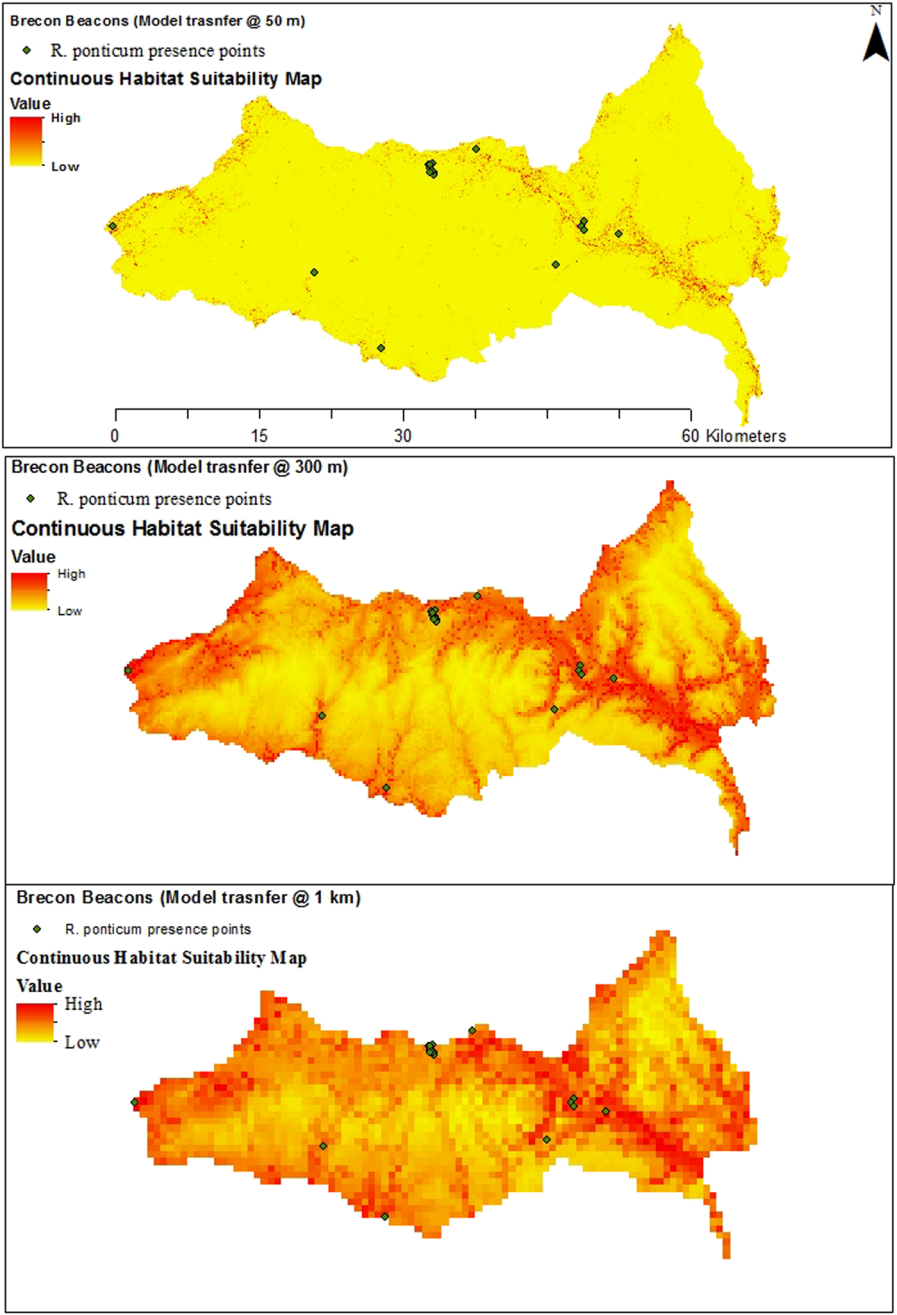
*Rhododendron ponticum* habitat suitability maps at 1 km, 300 m and 50 m resolutions generated in ArcGIS 10.5 (ESRI, Redlands, CA, USA, www.esri.com). A spatial distribution model was trained in Snowdonia National Park and transferred to the Brecon Beacons National Park. Blue dots indicate verified occurrence records of the species.

Although our study suggests that our model was transferred more accurately at 300 m grain size, it is important to mention that even at 50 m grain size, the model was also transferred with considerable success (CBI = 0.77). From an invasive species management point of view, a habitat suitability map at 50 m grain size with a lower prediction accuracy could still be more acceptable than a map with a better predictive ‘hit rate’ but at a six times coarser grain size. As an example, we include habitat suitability maps generated by model transfer to the Brecon Beacons National Park at three contrasting grain sizes (Fig. 2). The land cover map legend is provided in Supplementary data S3.

### Bioclimatic variables in SDMs – an inevitable choice?

In the context of our results it appears that unnecessary or ‘customary’ use of bioclimatic variables without considering the species’ ecology negatively affects the predictive potential of a SDM. Including these bioclimatic variables almost always comes at a cost of reducing the grain size of other variables, such as topography and land cover. However, as climate is likely to be one of the determinant of a species’ fundamental niche, we suggest that expert knowledge of species’ ecology and an extensive review of the literature should be carried out before deciding whether or not to include climatic variables in a SDM. Naturally, when modelling large-scale distributions (continental or global) or if the objective is a temporal prediction, perhaps to account for climate change, there currently may not be many alternatives to a 1 km grain size bioclimatic variables at a global scale. Choice of predictor variables is also a matter of the research question. If researches are strictly interested in estimating climatic suitability or sensitivity, then the climatic variables become an appropriate choice. Our results strictly refer to cases where researchers might be interested in mapping species’ distribution with high accuracy using the best possible combination of all the available predictor variables.

### Limitations of the study and future recommendations

Our study suggests that a grain size smaller than 1 km should be preferred in SDM studies; however, models using finer grain size data should be trained and validated with carefully validated occurrence records. Training a model with predictor variables at very small grain size leads to a very specific species-habitat relationship and thus needs to be verified with accurate presence records. Our study modelled the distribution of *R*. *ponticum*, a habitat specialist species that showed a clear response to the changes in grain size. By contrast, generalist species may not be as sensitive to a change in grain size. Our study also suggests that there may not be a ‘gold standard’ for the grain size of predictor variables when it comes to model transferability across space. Ideally, transferring the model to another area requires the identification of optimum grain size by considering a range of grain sizes, perhaps on a sub-set of available occurrence data. Also, we considered only a small area for model training and transferability possibly explaining why climatic variables contributed the least in our models. For SDMs over large spatial scale, climatic variables may have greater effect in determining the distribution of species. In this study, we have only used two evaluation tools (AUC & CBI) which hint that the model with higher values might be better than the rest. For future studies we recommend applying more robust statistics to evaluate the significance of difference between modelling scenarios.

## Electronic supplementary material

Review of literature

Analysis of Variables Contribution to Maxent-based Models

Response curves of Maxent based models and legends for land cover categories
