# Supplementary material for: Species distribution model transferability and model grain size – finer may not always be better
Source: Sci Rep. 2018 May 8;8:7168. doi: 10.1038/s41598-018-25437-1 (PMC5940916; doi:10.1038/s41598-018-25437-1)
Supplement: Supplementary file 1 — Review of literature [file 41598_2018_25437_MOESM1_ESM.docx]

**Species distribution model transferability and model grain size – finer may not always be better.**

Syed Amir Manzoor^1*^, Geoffrey Griffiths^2^ and Martin Lukac^1, 3^

^1^ School of Agriculture, Policy and Development, University of Reading, Reading, U.K.

^2^ Department of Geography and Environmental Sciences, University of Reading, Reading, UK

^3^ Faculty of Forestry and Wood Sciences, Czech University of Life Sciences Prague, Czech Republic

**Review of Literature**

Research papers published on MAXENT based Species Distribution Modelling in the years 2016-2017.

*Third column: ‘Climate data at 1 km resolution (or higher)’ – Studies which included bioclimatic variables

*Fourth column: ‘Finer resolution Non-Climatic variables’ – Studies which included Non-climatic variables which are available at a resolution finer than variables in third column.

*Fifth Column – NA: Studies which either had Climatic or Non-climatic variables. This classification is therefore *Not Applicable* to such studies.

| **No** | **Title of the research paper** | **Climate data at 1 km resolution (or higher)** | **Finer Resolution Non-climatic variables** | **Most important predictor variables (Climatic/Non-climatic/NA)** |
| --- | --- | --- | --- | --- |
| 1 | The influence of climate change on an endangered riparian plant species: The root of riparian Homonoia | Yes | Yes | Non-climatic |
| 2 | The importance of herbivore density and management as determinants of the distribution of rare plant specie | Yes | Yes | Non-climatic |
| 3 | Revealing areas of high nature conservation importance in a seasonally dry tropical forest in Brazil: Combination of modelled plant diversity hot spots and threat patterns | Yes | Yes | Climatic |
| 4 | Modeling and mapping the current and future climatic-niche of endangered Himalayan musk deer | Yes | No | NA |
| 5 | Spatial modelling of congruence of native biodiversity and potential hotspots of forest invasive species (FIS) in central Indian landscape | Yes | Yes | Non-climatic |
| 6 | Predicting current and future disease outbreaks of *Diplodia sapinea* shoot blight in Italy: species distribution models as a tool for forest management planning | Yes | Yes | Non-climatic |
| 7 | Species distribution modeling for wildlife management: Ornamental butterflies in México | Yes | No | NA |
| 8 | Environmental stress effects on reproduction and sexual dimorphism in the gynodioecious species *Silene acauli* | Yes | No | NA |
| 9 | Endemic grasshopper species distribution in an agro-natural landscape of the Cape Floristic Region, South Africa | Yes | Yes | Non-climatic |
| 10 | Factors affecting seasonal habitat use, and predicted range of two tropical deer in Indonesian rainforest | Yes | Yes | Non-climatic |
| 11 | Biodiversity hotspots and conservation gaps in Iran | Yes | Yes | Non-climatic |
| 12 | Identifying biodiversity hotspots for threatened mammal species in Iran | Yes | Yes | Non-climatic |
| 13 | Potential distributional changes of invasive crop pest species associated with global climate change | Yes | Yes | Climatic |
| 14 | Habitat distribution modelling to identify areas of high conservation value under climate change for *Mangifera sylvatica*Roxb. of Bangladesh | Yes | No | NA |
| 15 | Dynamic response of East Asian Greater White-fronted Geese to changes of environment during migration: Use of multi-temporal species distribution model | Yes | Yes | Non-climatic |
| 16 | A bird's view of new conservation hotspots in China | Yes | Yes | Non-climatic |
| 17 | Probabilistic assessment of high concentrations of particulate matter (PM_10_) in Beijing, China | Yes | Yes | Non-climatic |
| 18 | Mapping priorities for conservation in Southeast Asia | Yes | Yes | Climatic |
| 19 | Influence of environmental factors on the distribution of *Calymperes* and *Syrrhopodon* (Calymperaceae, Bryophyta) in the Atlantic Forest of Northeastern Brazil | Yes | Yes | Climatic |
| 20 | Field validation of an invasive species Maxent model | No | Yes | NA |
| 21 | Maxent modeling for predicting the potential distribution of endangered medicinal plant (*H. riparia* Lour) in Yunnan, China | Yes | Yes | Non-climatic |
| 22 | Using species distribution models to assess the importance of Egypt's protected areas for the conservation of medicinal plants | Yes | Yes | Climatic |
| 23 | Landscape to site variations in species distribution models for endangered plants | Yes | Yes | Non-climatic |
| 24 | Detecting the richness and dissimilarity patterns of Theaceae species in southern China | Yes | Yes | Climatic |
| 25 | Predicting distribution of major forest tree species to potential impacts of climate change in the central Himalayan region | Yes | Yes | Non-climatic |
| 26 | Predicting the probable distribution and threat of invasive *Mimosa diplotricha* Suavalle and *Mikania micrantha* Kunth in a protected tropical grassland | Yes | Yes | Climatic |
| 27 | Impacts of the spatial scale of climate data on the modeled distribution probabilities of invasive tree species throughout the world | Yes | No | NA |
| 28 | Efficacy of conservation strategies for endangered oriental white storks (*Ciconia boyciana*) under climate change in Northeast China | Yes | Yes | Climatic |
| 29 | Modeling impacts of future climate on the distribution of Myristicaceae species in the Western Ghats, India | Yes | No | NA |
| 30 | Surrogate species protection in Bolivia under climate and land cover change scenarios | Yes | No | NA |
| 31 | Modeling the distributions of useful woody species in eastern Burkina Faso | Yes | Yes | Non-climatic |
| 32 | Effects of climate change on the future distributions of the top five freshwater invasive plants in South Africa | Yes | No | NA |
| 33 | Spatial distribution of dry forest orchids in the Cauca River Valley and Dagua Canyon: Towards a conservation strategy to climate change | Yes | Yes | Non-climatic |
| 34 | Geographical boundary and climatic analysis of *Pinus tabulaeformis* in China: Insights on its afforestation | Yes | Yes | Climatic |
| 35 | Niche breadth and the implications of climate change in the conservation of the genus *Astrophytum* (Cactaceae) | Yes | No | NA |
| 36 | Niche constraints to the northwards expansion of the common genet (*Genetta genetta,* Linnaeus 1758) in Europe | Yes | Yes | Climatic |
| 37 | Climate change and the distribution and conservation of the world's highest elevation woodlands in the South American Altiplano | Yes | No | NA |
| 38 | Preventing extinction and improving conservation status of Vanilla borneensis Rolfe—A rare, endemic and threatened orchid of Assam, India | No | Yes | NA |
| 39 | Influence of land use and meteorological factors on the spatial distribution of *Toxocara canis* and *Toxocara cati* eggs in soil in urban areas | Yes | Yes | Non-climatic |
| 40 | Species distribution modeling for wildlife management: Ornamental butterflies in México | Yes | No | NA |
| 41 | Impact of climate and host availability on future distribution of Colorado potato beetle | Yes | No | NA |
| 42 | Mapping the climatic suitable habitat of oriental arborvitae (Platycladus orientalis) for introduction and cultivation at a global scale | Yes | No | NA |
| 43 | Prediction of the potential geographic distribution of the ectomycorrhizal mushroom Tricholoma matsutake under multiple climate change scenarios | Yes | Yes | Non-climatic |
| 44 | Climate change and the ash dieback crisis | Yes | No | NA |
| 45 | Habitat mapping as a tool for water birds conservation planning in an arid zone wetland: The case study Hamun wetland | No | Yes | NA |
| 46 | Potential distribution of *Ursus americanus* in Mexico and its persistence: Implications for conservation | No | Yes | NA |
| 47 | The distribution of deep-sea sponge aggregations in the North Atlantic and implications for their effective spatial management | No | Yes | NA |
| 48 | Current and future suitability of wintering grounds for a long-distance migratory raptor | Yes | Yes | Non-climatic |
| 49 | Distribution Modeling of three screwworm species in the ecologically diverse landscape of North West Pakistan | Yes | Yes | Non-climatic |
| 50 | Predicting impacts of climate change on habitat connectivity of *Kalopanax septemlobus* in South Korea | Yes | Yes | Climatic |
| 51 | Vulnerability to climate change of cocoa in West Africa: Patterns, opportunities and limits to adaptation | Yes | No | NA |
| 52 | Priority areas for conservation of beach and dune vegetation of the Mexican Atlantic coast | Yes | No | NA |
| 53 | Seasonal habitat suitability modeling and factors affecting the distribution of Asian Houbara in East Iran | Yes | Yes | Non-climatic |
| 54 | Kamala tree as an indicator of the presence of Asian elephants during the dry season in the Shivalik landscape of northwestern India | Yes | Yes | Climatic |
| 55 | The worrying future of the endemic flora of a tropical mountain range under climate change | Yes | No | NA |
| 56 | Taxonomy and ecological niche modeling: Implications for the conservation of wood partridges (genus *Dendrortyx*) | Yes | No | NA |
| 57 | Climate change fosters the decline of epiphytic *Lobaria* species in Italy | Yes | No | NA |
| 58 | Landscape to site variations in species distribution models for endangered plants | Yes | Yes | Non-climatic |
| 59 | Performance of one-class classifiers for invasive species mapping using airborne imaging spectroscopy | No | Yes | NA |
