# Supplementary material for: Species distribution model transferability and model grain size – finer may not always be better
Source: Sci Rep. 2018 May 8;8:7168. doi: 10.1038/s41598-018-25437-1 (PMC5940916; doi:10.1038/s41598-018-25437-1)
Supplement: Supplementary file 2 — Analysis of Variables Contribution to Maxent-based Models [file 41598_2018_25437_MOESM2_ESM.docx]

**Species distribution model transferability and model grain size – finer may not always be better.**

Syed Amir Manzoor^1*^, Geoffrey Griffiths^2^ and Martin Lukac^1, 3^

^1^ School of Agriculture, Policy and Development, University of Reading, Reading, U.K.

^2^ Department of Geography and Environmental Sciences, University of Reading, Reading, UK

^3^ Faculty of Forestry and Wood Sciences, Czech University of Life Sciences Prague, Czech Republic

**Supplementary Material**

**Analysis of Variables Contribution to Maxent-based Models**

**Permutation Importance of variables - Model VS-3**

| **Variable** | **Permutation importance** |
| --- | --- |
| Altitude | 87.8 |
| Land cover | 5.9 |
| Distance from water channels | 5.4 |
| Aspect | 0.5 |
| Slope | 0.4 |

**­­­­­­­­­­­ Permutation Importance of variables - Model VS-2**

| **Variable** | **Permutation importance** |
| --- | --- |
| Altitude | 88.7 |
| Land cover | 4.7 |
| Distance from water channels | 2.5 |
| Aspect | 3.8 |
| Slope | 0.3 |

**Permutation Importance of variables - Model VS-1**

| **Variable** | **Permutation importance** |
| --- | --- |
| Altitude | 60 |
| Land cover | 14.4 |
| Distance from water channels | 14.1 |
| bio_3 | 0.3 |
| bio_2 | 1.7 |
| Aspect | 1 |
| bio_9 | 1.4 |
| Slope | 2.3 |
| bio_15 | 4.9 |
