# Supplementary material for: Species distribution model transferability and model grain size – finer may not always be better
Source: Sci Rep. 2018 May 8;8:7168. doi: 10.1038/s41598-018-25437-1 (PMC5940916; doi:10.1038/s41598-018-25437-1)
Supplement: Supplementary file 3 — Response curves of Maxent based models and legends for land cover categories [file 41598_2018_25437_MOESM3_ESM.docx]

**Species distribution model transferability and model grain size – finer may not always be better.**

Syed Amir Manzoor^1*^, Geoffrey Griffiths^2^ and Martin Lukac^1, 3^

^1^ School of Agriculture, Policy and Development, University of Reading, Reading, U.K.

^2^ Department of Geography and Environmental Sciences, University of Reading, Reading, UK

^3^ Faculty of Forestry and Wood Sciences, Czech University of Life Sciences Prague, Czech Republic

**Response curves**

These curves show how each environmental variable affects the Maxent prediction. The curves show how the predicted probability of presence changes as each environmental variable is varied, keeping all other environmental variables at their average sample value. The curves show the mean response of the 25 replicate Maxent runs (red) and and the mean +/- one standard deviation (blue, two shades for categorical variables).

1. **Model at 1 km**


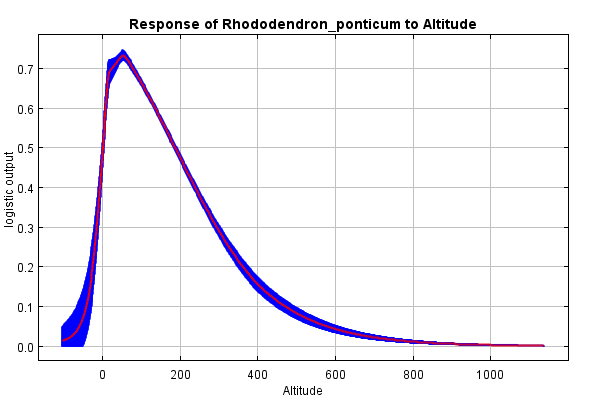

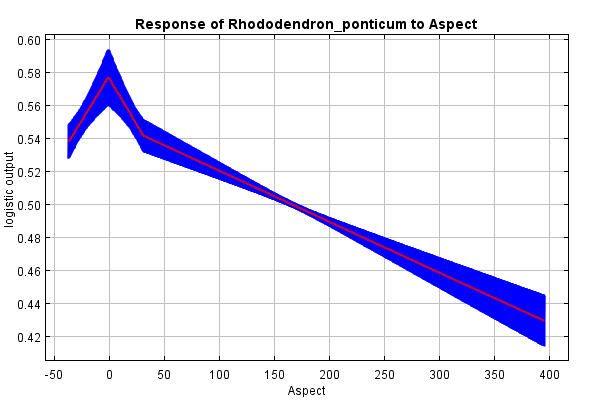


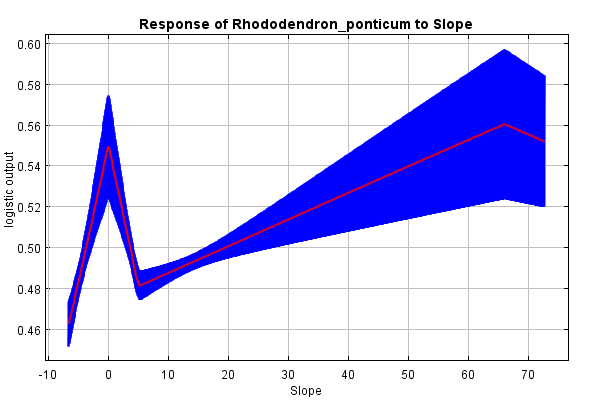

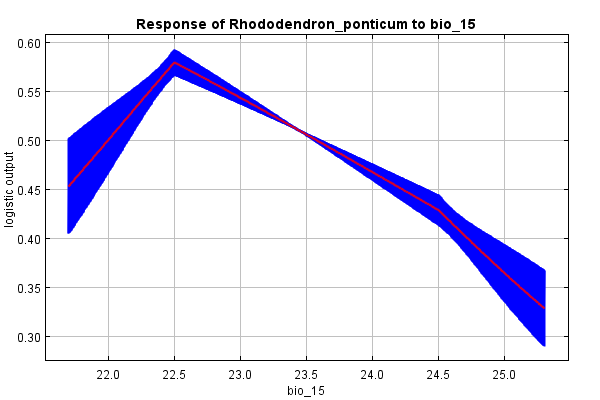


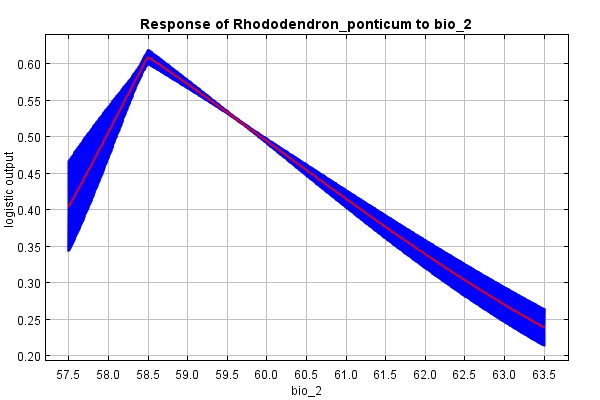

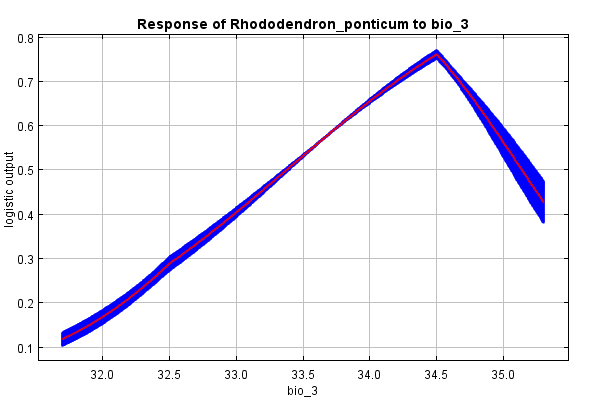


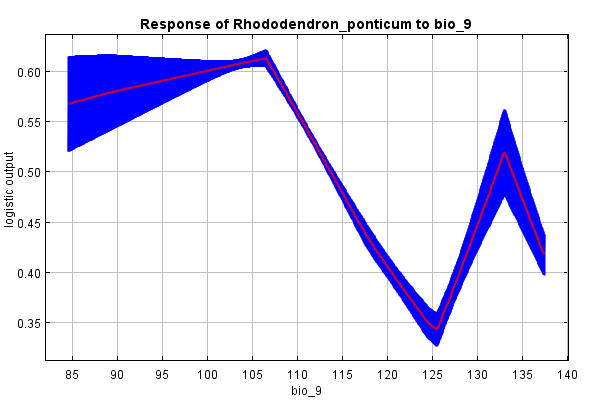

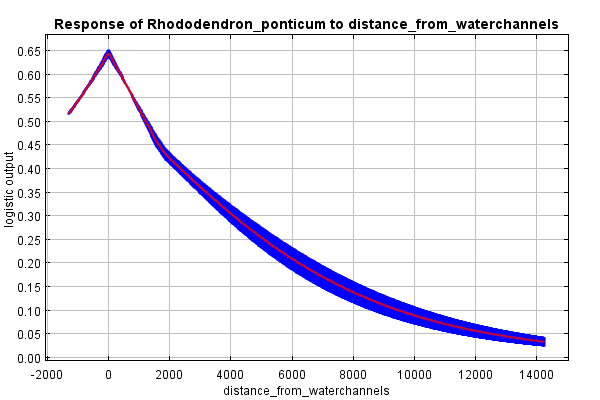


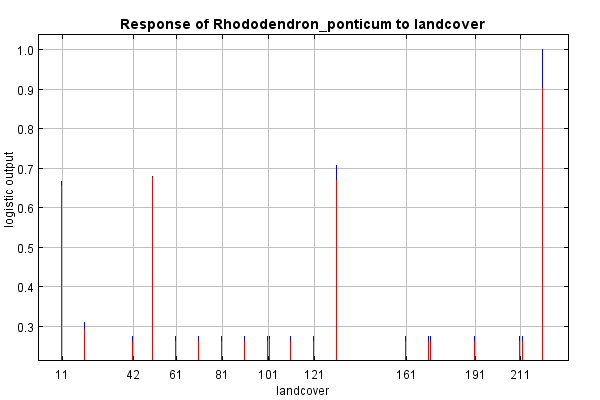


1. **Model at 300 m**


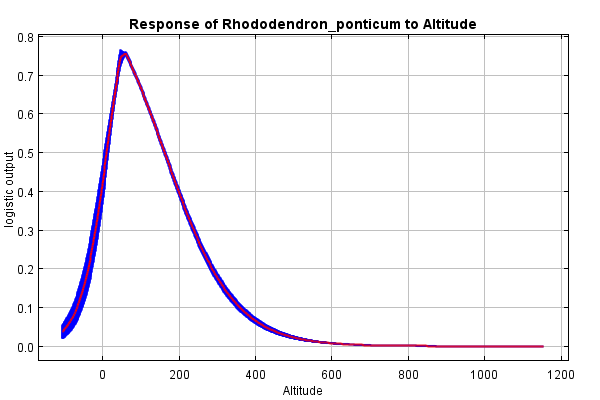

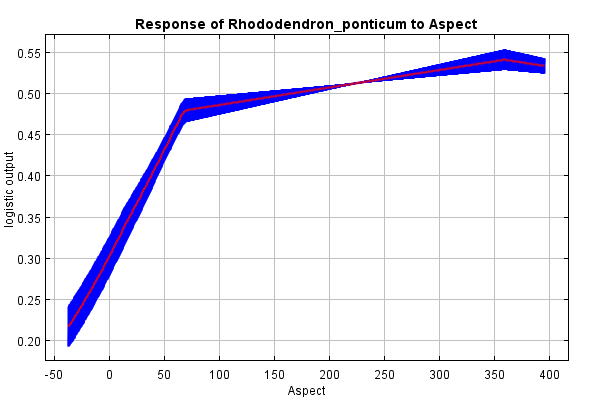


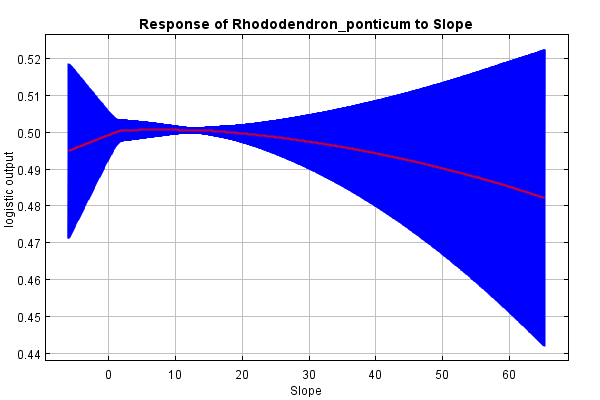

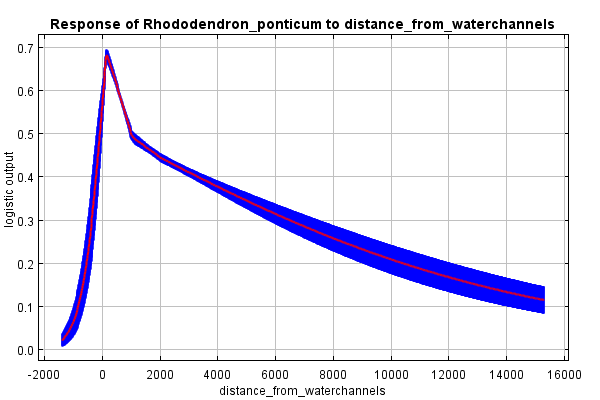


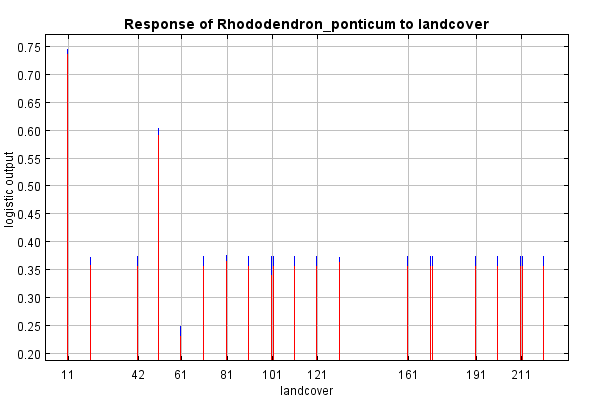


1. **Model at 50 m**


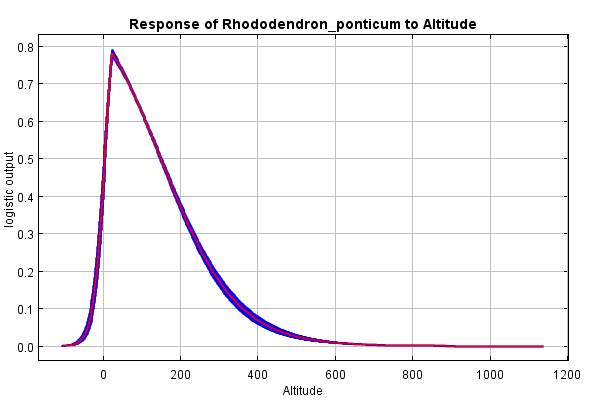

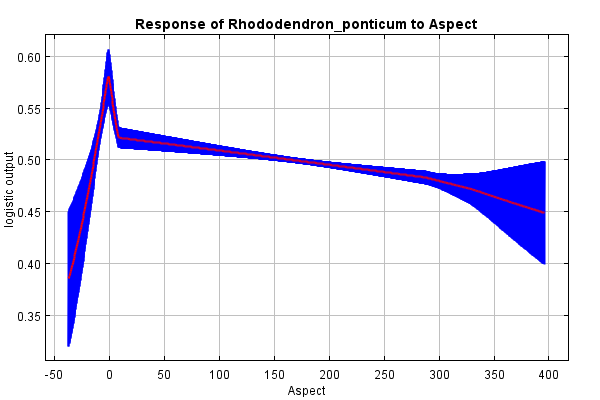


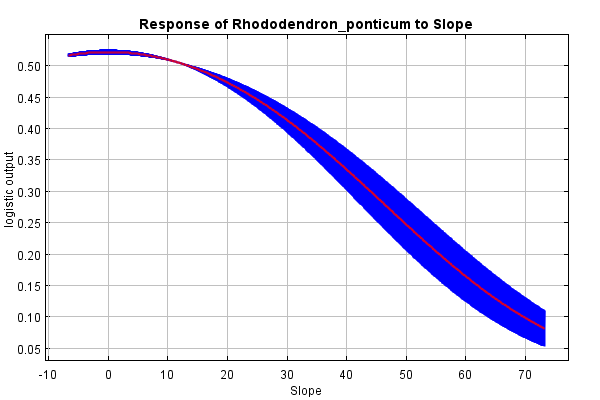

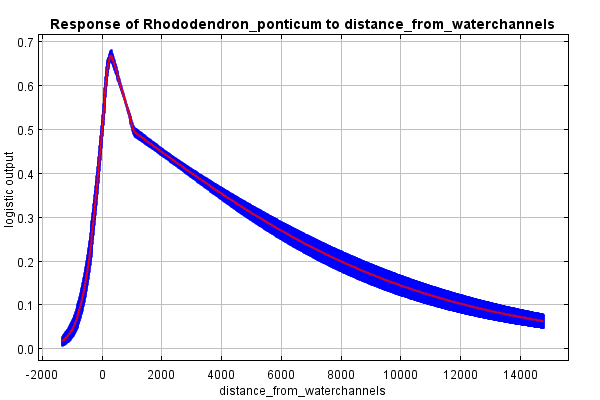


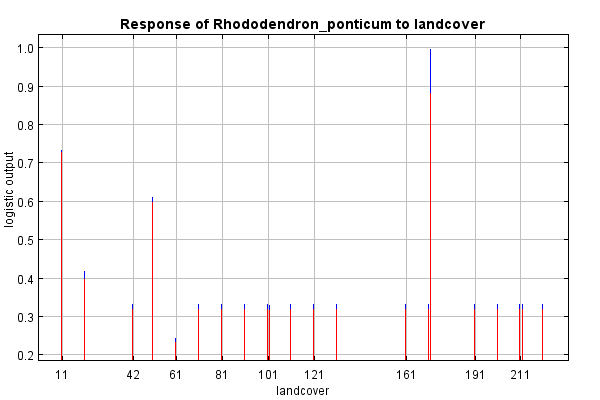


**Legend for Land cover categories**

| **11** | **Broad leaved woodland** |
| --- | --- |
| **21** | **Coniferous woodland** |
| **41** | **Arable cereals** |
| **42** | **Arable horticulture** |
| **43** | **Non Rotational Horticulture** |
| **51** | **Improved Grassland** |
| **52** | **Setaside Grassland** |
| **61** | **Neutral Grassland** |
| **71** | **Calcareous grassland** |
| **81** | **Acid grassland** |
| **91** | **Bracken** |
| **101** | **Dense dwarf shrub heath** |
| **102** | **Open dwarf shrub heath** |
| **111** | **Fen, marsh, swamp** |
| **121** | **Bog** |
| **131** | **Inland water** |
| **151** | **Montane habitat** |
| **161** | **Inland rock** |
| **171** | **Sub urban/rural developed** |
| **172** | **Continuous urban** |
| **181** | **Supra-littoral rock** |
| **191** | **Supra-littoral sediment** |
| **201** | **Littoral rock** |
| **211** | **Littoral sediment** |
| **212** | **Saltmarsh** |
| **221** | **Sea/Estuary** |
